# Human Imprinted Chromosomal Regions Are Historical Hot-Spots of Recombination

**DOI:** 10.1371/journal.pgen.0020101

**Published:** 2006-07-07

**Authors:** Ionel Sandovici, Sacha Kassovska-Bratinova, Joe E Vaughan, Rae Stewart, Mark Leppert, Carmen Sapienza

**Affiliations:** 1 Fels Institute for Cancer Research and Molecular Biology, Temple University School of Medicine, Philadelphia, Pennsylvania, United States of America; 2 College of Science and Technology, Temple University, Philadelphia, Pennsylvania, United States of America; 3 Eccles Institute of Human Genetics, and Department of Human Genetics, University of Utah, Salt Lake City, Utah, United States of America; 4 Department of Pathology and Laboratory Medicine, Temple University School of Medicine, Philadelphia, Pennsylvania, United States of America; North Carolina State University, United States of America

## Abstract

Human recombination rates vary along the chromosomes as well as between the two sexes. There is growing evidence that epigenetic factors may have an important influence on recombination rates, as well as on crossover position. Using both public database analysis and wet-bench approaches, we revisited the relationship between increased rates of meiotic recombination and genome imprinting. We constructed metric linkage disequilibrium (LD) maps for all human chromosomal regions known to contain one or more imprinted genes. We show that imprinted regions contain significantly more LD units (LDU) and have significantly more haplotype blocks of smaller sizes than flanking nonimprinted regions. There is also an excess of hot-spots of recombination at imprinted regions, and this is likely to do with the presence of imprinted genes, per se. These findings indicate that imprinted chromosomal regions are historical “hot-spots” of recombination. We also demonstrate, by direct segregation analysis at the 11p15.5 imprinted region, that there is remarkable agreement between sites of meiotic recombination and steps in LD maps. Although the increase in LDU/Megabase at imprinted regions is not associated with any significant enrichment for any particular sequence class, major sequence determinants of recombination rates seem to differ between imprinted and control regions. Interestingly, fine-mapping of recombination events within the most male meiosis–specific recombination hot-spot of Chromosome 11p15.5 indicates that many events may occur within or directly adjacent to regions that are differentially methylated in somatic cells. Taken together, these findings support the involvement of a combination of specific DNA sequences and epigenetic factors as major determinants of hot-spots of recombination at imprinted chromosomal regions.

## Synopsis

Now that the finished reference sequence of the human genome is available, focus has shifted towards understanding fundamental aspects of its functions. Meiotic recombination between maternal and paternal chromosomes serves an important mechanistic and evolutionary role in the transmission of the genome. Although significant progress has been made towards fine-mapping meiotic recombination events along human chromosomes, the characterization of factors that influence the position and frequency of crossovers remains a challenge. These authors have used data generated by the International HapMap Project as well as experimental analysis of a collection of three-generation Centre d'Etude du Polymorphisme Humain (CEPH) families, to show that chromosomal regions containing imprinted genes (i.e., genes transcribed only from one allele in a parent-of-origin–specific manner) exhibit higher rates of meiotic recombination than nonimprinted chromosomal regions. This characteristic is common for all major human populations. The major sequence determinants of recombination rates are likely to be different at imprinted and nonimprinted regions. Moreover, epigenetic modifications associated with imprinted regions may play an important role in increasing the frequency of meiotic crossovers and determining their position. Taken together these results suggest that a complex series of factors control meiotic recombination in the human.

## Introduction

In the human, as well as in other eukaryotes, sites of recombination are not randomly distributed along the chromosomes because of the presence of numerous hot-spots and cold-spots of recombination [1]. Little is known about the rules that govern the distribution of recombination events, although age, sex, DNA sequence, chromatin structure, chromosomal location, and chromosome sizes have been shown to be important [2,3]. In addition, we have suggested [4] that there may be a mechanistic link between the processes of imprinting and recombination.

Sex-specific recombination hot-spots have been identified in the vicinity of two human imprinted regions: 11p15.5 and 15q11–q13 [5,6], as well as around the *Igf2* locus in sheep [7]. More recently, Lercher and Hurst [8] have shown that most, if not all, imprinted chromosomal regions in the human genome have unusually high (and possibly sex-specific) recombination rates. These last authors used meiotic mapping data from the deCODE map [9] which has a resolution of about 1 cM. However, this window is considerably larger than most of the chromosomal regions containing imprinted genes, and the limited resolution of the map with respect to the size of imprinted regions has the potential to make their findings conservative.

Recombination rates may also be inferred from genotype information collected on populations of unrelated individuals, by examining patterns of linkage disequilibrium (LD). Although there are many factors that may influence the extent of LD (such as mutation, selection, and genetic drift), recombination is the main determinant of LD patterns across the genome [10]. LD and recombination are negatively correlated: A cold-spot for LD is a hot-spot for recombination and vice versa [9,11–16]. We used LD data from the International HapMap Project [17,18] to infer the recombination history at regions containing known imprinted genes.

In an attempt to better understand the relationship between imprinting and recombination, we focused further attention on the imprinted region at human Chromosome 11p15.5, using a collection of archival DNA samples from 45 three-generation Centre d'Etude du Polymorphisme Humain (CEPH) families and constructed both genetic and LD maps. We attempted to determine which factors are responsible for the breakdown of LD and, indirectly, which factors result in an increase of recombination rates at imprinted chromosomal regions.

## Results

### Analysis of Metric LD Maps at Human Chromosomal Regions Containing Known Imprinted Genes

We retrieved genotypes, allele frequencies and *D′* values from the HapMap Project database (http://www.hapmap.org) for the four major populations (CEU—CEPH families with European ancestry; HCB—Han Chinese in Beijing, China; JPT—Japanese in Tokyo, Japan; and YRI—Yoruba in Ibadan, Nigeria). According to the public release #19 (October 2005), there were nearly 6 million genotyped single nucleotide polymorphisms (SNPs) available across the entire genome, with a mean spacing of about 0.5 kilobases (kb).

In order to quantify the relationship between LD and recombination over all imprinted regions, we used the recently described methodology of calculating metric linkage disequilibrium units (LDUs) between pairs of SNPs [12], and constructed metric LD maps for all regions containing known imprinted genes (see Materials and Methods). The theoretical framework for constructing LD maps is based on the Malecot model [19] that describes the exponential decay of LD with time in descendants of a hypothetical starting population showing complete LD, with the rate of decay governed by recombination rate. We identified 17 regions containing one or several imprinted genes (http://www.geneimprint.com; see Tables S1–S4). Because recombination rates correlate strongly with LDUs when large-scale bins (1–5 megabases [Mb]) are used [16], we analyzed windows of about 1 Mb (for isolated imprinted genes) or more (for clusters of genes). These windows were obtained by centering first on the imprinted gene or genes and then by zooming out until the established sizes were achieved.

The total of the imprinted bins, corresponding to approximately 26 Mb, comprise 596 LDUs for the JPT population, 605 LDUs for the HCB population, 683 LDUs for the CEU population, and 1,273 LDUs for the YRI population (see Tables S1–S4). These correspond to excesses of LDUs of 39.5%, 31%, 44.7%, and 99%, respectively, as compared with regions of equal size chosen randomly from the genome, as extrapolated from an analysis on three human chromosomes [16].

**Figure 1:**
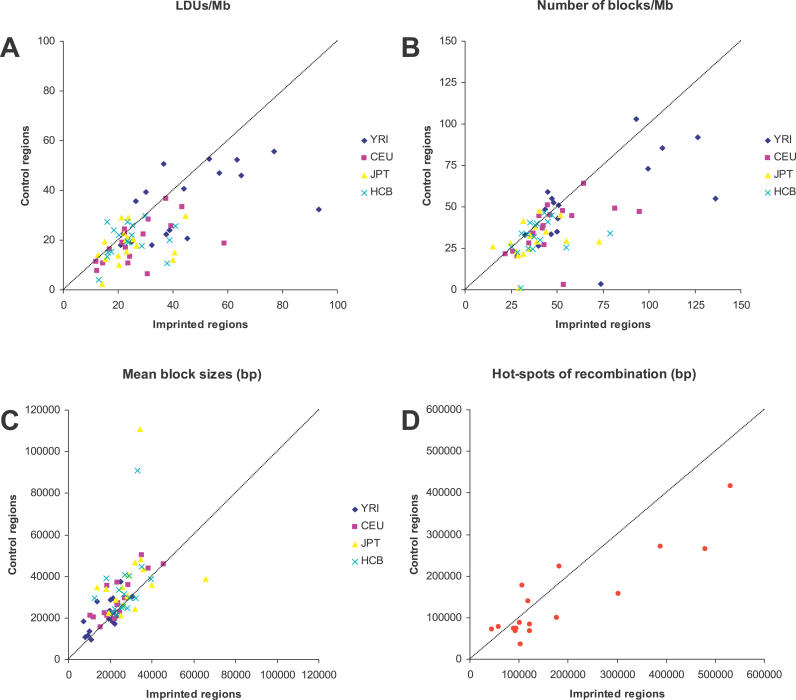
LD Analysis at Human Chromosomal Regions Containing Imprinted Genes (A) Comparison of LDU values at imprinted versus control regions. Each LDU/Mb value obtained for a given imprinted bin was plotted against the LDU/Mb value of the corresponding control region. Note that most of the imprinted regions reach higher LDU values compared with their corresponding control regions (dotted line with slope 1 corresponds to virtual positions in cases with equal LDU/Mb values at imprinted and control regions). (B) The number of haplotype blocks/Mb is higher at imprinted genes compared with their corresponding control regions (dotted line indicates equal values). (C) The mean sizes of haplotype blocks are significantly smaller at imprinted regions versus flanking control regions (dotted line indicates equal sizes). (D) There is a significant excess in the number of hot-spots of recombination at imprinted versus control regions (see text), and the total length of the hot-spots appears greater in imprinted regions than in control regions. Each value corresponds to the total length of all hot-spots of recombination (in base pairs [bp]) for a given imprinted or control region.

Because some of the imprinted regions have values lower than the genome-wide means, we also considered whether imprinted regions contain more LDUs than their flanking sequences. For this analysis, we used flanking control bins of size equal to each imprinted region. Each control region was comprised of two bins (one telomeric and one centromeric) of sizes equal to half of the imprinted region they flank. As a result, 26 Mb of control regions were analyzed. We performed paired Student *t* tests for pairs of imprinted–control regions. As seen in Figure 1A, imprinted regions have significantly higher metric LD values per Mb (*p* < 0.0001). At the single-locus level, eight out of the 17 imprinted regions have higher LDU values than their corresponding control regions in all four populations, four imprinted regions have higher LDU values in three of the four populations, four imprinted loci have higher LDU values in only two populations, whereas one locus *(L3MBTL)* has lower LDU values in all four populations. We then calculated the number of haplotype blocks, defined as continuous chromosomal regions with ΔLDU = 0 [20], for both imprinted and control regions. We found that imprinted regions have significantly more haplotype blocks per Mb than the control regions (*p* < 0.0001) (Figure 1B), and that the mean block sizes are significantly smaller (*p* = 0.0003) (Figure 1C). These differences remain significant when considering each population separately, except for the number of haplotype blocks in the CEU population and for the mean block size in the JPT population (see Tables S1–S4).

The HapMap Project also provides the location of recombination hot-spots estimated from Phase I HapMap data (release 16a) using the coalescent method described in McVean et al. [13] and Winckler et al. [15]. Based on these data, there are 254 hot-spots of recombination at imprinted regions versus 209 hot-spots of recombination at control regions (sign test *p* = 0.04). Although the resolution of these recombination hot-spots does not reach the same level of precision as in methods based on sperm typing [11], the length of recombination hot-spots at imprinted regions is significantly greater than those in paired control regions (paired Student *t* test *p* = 0.0293) (Figure 1D). (The minimum width of a detectable hotspot is 2 kb, similar to that observed by sperm typing, but this level of resolution will only be true in the rare instance in which the 2-kb region will have the maximum recombination in a 200-kb window and adjacent 2-kb regions are not within a factor of two of this maximum—see HapMap Web site [http://www.hapmap.org].) This excess of recombination hot-spots seems to be linked with the presence of imprinted genes at these regions. At control regions, we identified 91 hot-spots of recombination associated with one of 361 genes. At imprinted regions, we identified 112 hot-spots of recombination associated with one or several of 415 genes (*χ^2^* test between genes at imprinted versus genes at control regions *p* = 0.666, suggesting that the difference in the number of hot-spots is not simply due to higher gene density in imprinted regions). However, 27 of these hot-spots were located inside the 41 genes known to be transcriptionally imprinted in human and shown on Haploview (*χ^2^* test between hot-spots at imprinted genes versus hot-spots at all genes from imprinted regions *p* = 0.0007), suggesting that the difference in the number of hot-spots is due to the presence of imprinted genes, per se.

### Analysis of Sex-Specific Meiotic Recombination Rates at the 11p15.5 Human Imprinted Cluster

We extended an earlier analysis of meiotic recombination events at the 11p15.5 human imprinted cluster [5] by using DNA samples from a panel of 45 three-generation families and a higher density of markers. We began by genotyping two highly informative microsatellites: D11S2071 (position 0.236 Mb on Chromosome 11) and D11S1760 (position 5.310 Mb) which allowed us to identify 390 informative male meioses and 386 informative female meioses. This region contains most of the imprinted genes described at the 11p15.5 cluster, except for *ZNF215,* which is located 1.6 Mb further toward the centromere (position 6.90–6.94 Mb).

**Figure 2:**
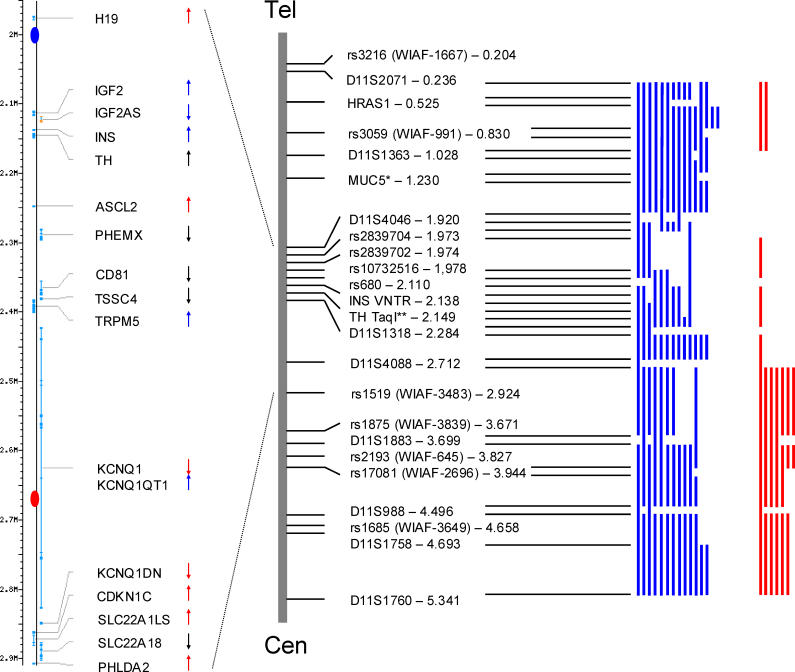
Distribution of Recombination Events at Human 11p15.5 Imprinted Cluster Positions of markers used for mapping recombinants in this region are indicated in Mb from the telomeric end (Tel) of the short arm. Imprinted genes are shown on the left side of the figure. Arrows correspond to direction and parental-specific origin of transcription: blue are paternally transcribed genes, red are maternally expressed genes, and black are genes with biallelic expression or unknown imprinting status. The two known germline imprints at this locus are shown by colored oval shapes on the left side of the figure: the blue oval corresponds to the paternally methylated *IGF2*/*H19* DMR and the red oval corresponds to the maternally methylated *KCNQ1OT1* DMR. Each vertical bar on the right side of the figure corresponds to a meiotic recombination event, delimitated by the nearest informative markers: Labeled in blue are crossovers in paternal meiosis, and labeled in red are recombinations in maternal meiosis. An asterisk (*) represents an unidentified polymorphism found at *MUC5B* locus, and double asterisks (**) indicate an unidentified TaqI polymorphism found at *TH* locus (genotypes available through CEPH database—see Materials and Methods).

Pedigree analysis showed the presence of 73 paternal recombination events as well as 19 maternal recombination events (sex-specific recombination difference *p* < 0.0001, Fisher's exact test). We attempted to fine-map these crossovers using an additional panel of 22 markers (microsatellites, minisatellites, and SNPs—see Materials and Methods). As shown in Figure 2, we found that the maximum bias in male meiotic recombination (46 or 47 paternal recombinations versus four or five maternal recombinations) is telomeric to D11S4088, corresponding to the region that contains the majority of imprinted genes from the 11p15.5 cluster. This sex difference is the result of a more than 5-fold increase over the average male recombination rate (4.55 cM/Mb versus the male-specific genome-wide average of 0.81 cM/Mb; [9]), as well as a 3-fold decrease in female recombination rate (to 0.5 cM/Mb versus the female-specific genome-wide average of 1.4 cM/Mb; [9]). There are 26 or 27 recombinations in male meiosis and 14 or 15 recombinations in female meiosis that occur centromeric to D11S4088. The rate of recombination in the interval between D11S4088 and D11S1760 remains higher than expected in male meiosis (about 2.5 cM/Mb), but becomes normal in female meiosis (1.5cM/Mb). However, the sex differences in this interval are not significant.

### Fine-Mapping of Recombination Sites in the 11p15.5 Region

Because differential DNA methylation of maternal and paternal alleles is a characteristic feature of imprinted regions, we attempted to map more precisely the locations of crossovers with respect to differentially methylated regions (DMRs) in the 11p15.5 imprinted clusters. There are a number of well-characterized DMRs in this region [21–23], and two are strongly associated with differential transcription [24,25]. The first DMR is between *IGF2* and *H19* (blue oval on left side of Figure 2) (http://www.ncbi.nlm.nih.gov/mapview) and the second is found in intron 10 of the *KCNQ1* gene, at the *KCNQ1OT1* promoter (red oval on left side of Figure 2) (http://www.ncbi.nlm.nih.gov/mapview).

Twenty of the 46 or 47 paternal recombinations telomeric to D11S4088 and one of the four or five maternal recombinations overlap one or the other of the two DMRs (Figure 2). Seven male recombination events and one female recombination event overlap the *IGF2/H19* DMR whereas the *KCNQ1OT1* DMR is overlapped by 13 male recombinations and one female recombination. We localized (by genotyping additional SNPs; see Materials and Methods) one female recombination to a maximum interval of 29.6 kb (between a C/T polymorphism found at position 1,974,950, corresponding to rs11564733—identified by sequencing—and a C/G polymorphism corresponding to rs7924887, position 2,004,532—found at a TaqI site). Two additional paternal recombinations were mapped very close to the *IGF2*/*H19* DMR: one to a maximum interval of less than 5 kb, between rs4930125 (a G/T polymorphism at a MspI site—position 1,995,850) and rs4930001 (an A/G polymorphism at a TaqI site—position 2,000,443), and the second to an 8.7-kb interval between rs4930125 (position 1,995,850) and rs7924887 (position 2,004,532). Two paternal recombinations overlapped the *KCNQ1OT1* DMR when additional SNPs were genotyped; both of these events were located between D11S4088 (position 2.676 Mb) and rs2283202 (an A/G polymorphism found at an HpaII site—position 2,694,481).

### LD Analysis at Imprinted Human Chromosomal Region 11p15.5

**Figure 3:**
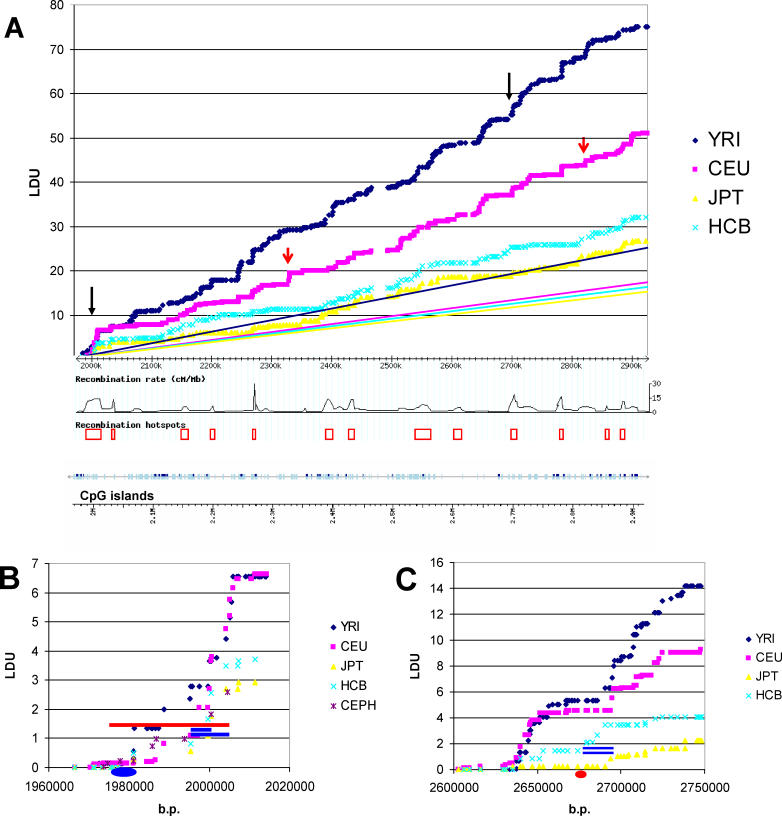
LD Analysis at Human 11p15.5 Imprinted Cluster (A) Population-specific metric LD map for about 1-Mb region containing imprinted genes at human 11p15.5 chromosome. Positions along the chromosome are shown in bp on the *x-*axis. Straight lines are representing the genome-wide slopes (LDU/Mb) corresponding to each population, as extrapolated from De La Vega et al. [16]. Note that LD extends less far in the region containing imprinted genes compared with a region of similar length from the rest of genome, in agreement with the interpretation of higher recombination in these areas (i.e., breakdown of LD has been converted to implied recombination rate and rendered graphically as red rectangular “hot spots”). Location of CpG islands in the region are depicted as shown in MapView; dark blue represents CpG islands larger than 500 bp, and light blue represents CpG islands over 200 bp. For both categories, G + C content is higher than 50% and the observed CpG/expected CpG content is higher than 0.6. The two black arrows correspond to the regions containing the primary germline imprints at *H19*/*IGF2* DMR (left arrow) and *KCNQ1OT1* DMR (right arrow), respectively. Both are located at regions exhibiting steps of LD and recombination hot-spots and are zoomed-in in (B) and (C). The red open arrows correspond to smaller steps, which are variable between populations and do not correspond with any hot-spot of recombination. (B) The metric LD map for the region containing *H19*/*IGF2* DMR using data from the four populations (HapMap) and the set of CEPH individuals analyzed in this study. The three horizontal bars correspond to recombinants mapped at this region, one in maternal meiosis (red) and two in paternal meioses (blue). The blue oval shape corresponds to the *H19*/*IGF2* DMR. (C) The metric LD map for the region containing *KCNQ1OT1* DMR using data from the four populations (HapMap). Two recombinants (horizontal blue bars) were mapped at this region in paternal meioses. The red oval shape corresponds to the *KCNQ1OT1* DMR.

In order to determine whether the particular sites of recombination we identified in the CEPH families corresponded to universal sites of LD breakdown, we used the HapMap Project database to retrieve genotypes, allele frequencies, and *D′* data for all four major populations available in the HapMap Project. We reconstructed LDU maps for these populations over a region of about 1 Mb at human Chromosome 11p15.5 (from rs1706879 at 1,966,471 to rs4758562 at 2,920,246), that contains most of the known imprinted genes in this region (except *ZNF215*) (Figure 3A).

The pattern of plateaus (corresponding to blocks of low haplotype diversity) and steps (regions of high haplotype diversity) observed and the height of each step is thought to reflect the recombination history of the region [26]. Indeed, as shown in Figure 3A, the most abrupt and conspicuous steps appear to be common to all four populations and have been interpreted to correspond to historical hot-spots of recombination, as indicated on the Haploview image of this region. Smaller steps in the region (e.g., red arrows in Figure 3A) seem to be more variable between populations and do not correspond to any obvious hot-spot of recombination and may suggest some population differences in characteristic sites of recombination or less common events that did not occur in all populations. Figure 3A also shows that higher recombination rates (cM/Mb) and the presence of hot-spots of recombination tend to associate with higher densities of CpG islands. Two major steps, common to all four populations, are found in the vicinity of the two imprinting centers (one centromeric to the *H19* gene and the other inside the *KCNQ1* gene; black arrows in Figure 3A). These are also the regions to which we fine-mapped several meiotic recombination events in the collection of CEPH samples (Figure 3B and 3C), confirming that LDU maps are efficient tools in the localization of recombination hot-spots [26].

**Figure 4:**
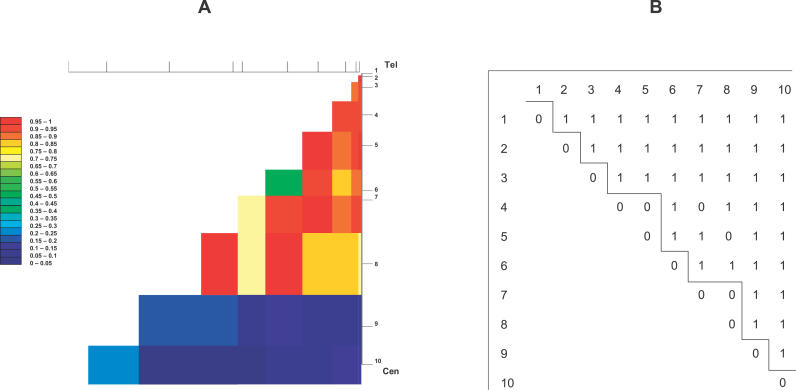
Pairwise LD Analysis and Pairwise FGT at the *H19*/*IGF2* DMR (A) Pairwise LD test between ten SNPs covering a 31-kb region containing *H19*/*IGF2* DMR shows a major breakdown of LD which corresponds to the LDU step shown in Figure 3B. Intensity of LD is coded in colors as shown. (B) Pairwise FGT between the same ten SNPs. A “1” indicates recombination between that pair of loci (all four gametes) and “0” indicates only three types of gametes (recombination between the two loci is uncertain). Considering that a historical recombination would break the haplotype inside of which it appeared, at least eight haplotype blocks could be identified. The ten markers used for both analyses in Figure 4A and 4B are the same as depicted in Figure 3B (CEPH)

We also used the collection of CEPH families to genotype ten SNPs in a region of about 30 kb that overlaps the imprinting center located centromeric of the *H19* gene (see Materials and Methods). We then constructed haplotypes based on pedigree analysis and used data obtained for founders (unrelated first-generation individuals) for further LD tests. In a panel of 278 first-generation individuals, we identified 52 unique haplotypes (out of 1,024 maximum theoretically possible). As the mutation rate in the human genome is very low (2.5 × 10^−8^ per site per generation) [27] relative to the number of generations since the most recent common ancestor of any two humans (of the order of 10^4^ generations), nearly every variable site in our genome results from a single historical mutational event. This assumption is likely to remain true even for mutational hot-spots such as CpG dinucleotides, in which the frequency of both transitions and transversions is known to be one order of magnitude higher [27]. This logic suggests that only 11 of the identified haplotypes could be explained by historical mutations (the “ancestral” haplotype plus ten additional that resulted from new mutations). The observed high diversity of haplotypes in this relatively small region indicates a very fragmented structure of the haplotype resulting from an increased frequency of meiotic recombination. This is supported by the LD analysis (Figure 4A) that shows a major breakdown of intermarker LD as well as the presence of a major step in the metric LD map (Figure 3B). Also, the four-gamete test (FGT) [28,29] shows that, with the exception of two pairs of SNPs (rs10732516 and rs2525886—3,245 base pairs (bp) apart; and rs3858516 and rs4384367—6,865 bp apart), at least one historical recombination event occurred between each of the loci analyzed (Figure 4B). All of these results support the hypothesis that this region is a historical hot-spot of recombination.

### Determinants of LDU at Imprinted and Control Regions

We attempted to identify genomic characteristics that could account for the LDU/Mb differences between imprinted and control regions. First, we used the CPGPLOT and CPGREPORT programs in EMBOSS [30] and the RepeatMasker program (see Materials and Methods) to compare the same pairs of imprinted and control regions described above (Table S1) for the presence of CpG islands, CpG dinucleotides, GC content and classes of repeats, and gene content (Table 1).

**Table 1:**
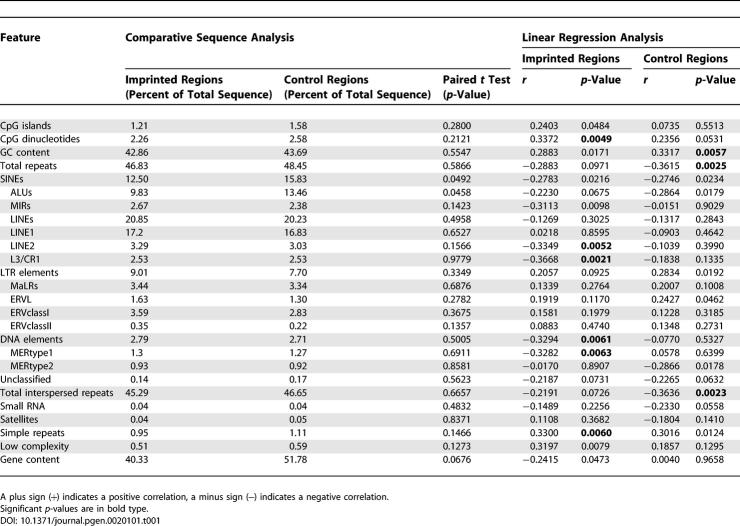
Summary of Comparative Sequence Analysis and Linear Regression Analysis of the Relationship between Sequence Features and LDU/Mb Ratios at Imprinted versus Corresponding Flanking Control Regions

We then attempted to determine whether any of these sequence classes could account for LDU differences between imprinted regions and their flanking control regions. We calculated LDU/Mb ratios for each bin and each population, and performed linear regression analysis with each of the DNA features shown in Table 1. After correcting for multiple tests (see Materials and Methods), we found that the sequence features that exhibit significant correlations at imprinted and control regions are mutually exclusive (Table 1). GC content is the only sequence feature that is significantly and positively correlated with LDU/Mb at control regions, as has been observed for the genome overall [16,20,31–34]. The combined sequence class “total repeats” (as well the interrelated parameter “total interspersed repeats”) shows a significant but negative correlation with LDU/Mb ratios at control regions. In contrast, the single largest positive correlation with LDU/Mb ratios at imprinted genes is with CpG dinucleotides. Simple repeats also exhibit positive correlation with LDU/Mb ratios, whereas LINE 2 and L3/CR1 repeats as well as DNA elements show significant negative correlation (Table 1).

We also analyzed several short DNA motifs that have been demonstrated to be enriched at hot-spots of recombination in a recent genome-wide analysis [35]. We have selected motifs that are strongly signaled within THE1B hot-spots or the top motifs from each of the 6-mer to 9-mer motifs. We found that all motifs included in Table 2, with the exception of TACTGTTC, are enriched at the 463 hot-spots of recombination within the analyzed regions (significant *χ^2^* tests for each of the six enriched motifs with *p* < 0.0001). We also analyzed their global distribution at imprinted and control regions after masking for repeats. There is no significant difference between imprinted and corresponding control regions for any of these elements. Further, we performed linear regression analysis between LDU/Mb ratios at each bin and the density of each of these DNA motifs per Mb. After correcting for multiple testing (see Materials and Methods), we found once more that sequence features that exhibit significant correlations at imprinted and control regions are mutually exclusive. It is noteworthy that the strongest positive correlation of LDU/Mb ratios at imprinted regions is with a DNA motif that contains a CpG dinucleotide.

**Table 2:**
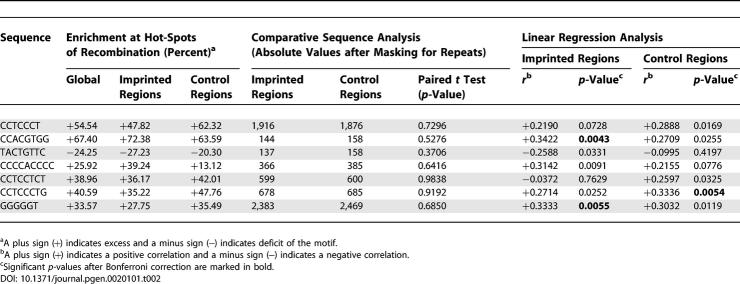
Distribution and Linear Regression Analysis of Short DNA Motifs Associated with Hot-Spots of Recombination

## Discussion

We used a combination of fine-structure meiotic mapping, haplotype analysis, and LD mapping to examine rates of recombination in imprinted regions of the human genome. Our fine-structure meiotic mapping results in the Chromosome 11p15.5–imprinted region agree with the studies of Paldi et al. [5] and Lercher and Hurst [8] that this region is a hot-spot of recombination in male meiosis. The main disadvantage of the LD mapping method used in our study in comparison with the meiotic mapping study of Lercher and Hurst [8] is the inability to determine sex-specific recombination rates in our study. Reciprocally, the use of LD maps has two main advantages. First, LD breakdown provides information that may be interpreted as repeated recombination events in a given region over many generations. Second, the high density of SNPs (mean distance between consecutive SNPs in this study is less than 0.5 kb) results in very high resolution in identifying regions with increased recombination rates. Areas with short blocks and large LDU steps coincide with recombination hot-spots, and conversely, regions with longer blocks and minor steps correspond to recombination cold-spots. We found that each imprinted region contains several regions with apparent increase in historical recombination rate, even for those regions where only one imprinted gene has been described so far. Several imprinted regions exhibit population-level differences in haplotype block size (e.g., “outlier” JPT and HCB points in Figure 1C), or some populations appear to lack LDU steps that are present in other populations (Figure 3C), suggesting the possibility of between-population variability in sites or rates of recombination. It will be interesting to determine whether these differences reflect similar differences in epigenetic marking between populations, as we have observed within the CEPH population [36].

One additional point suggested by the comparison of imprinted and control regions in Figure 1 is the correlation between indicators of recombination in the two regions. We observed a positive correlation for LDU/Mb (*r^2^* = 0.4252) and for number of haplotype blocks/Mb (*r^2^* = 0.4118). There is also a positive correlation for the mean size of haplotype blocks between imprinted and control regions (*r^2^* = 0.2797) as well as for the total length of recombination hot-spots per bin (*r^2^* = 0.8018). We interpret these findings as an indirect argument that the chromosomal position of any given region is a major determinant for crossover activity and that this might act at a large scale. Specific DNA sequences and possible epigenetic factors are then able to modulate the frequency of meiotic recombination in a given region (e.g., by increasing the number of hot-spots of recombination).

Imprinted chromosomal regions have two unusual characteristics in terms of meiotic recombination: They have unusually high recombination rates compared with their flanking regions, and at least some imprinted regions exhibit heterochiasmy (i.e., quantitative differences in recombination rate between the two sexes). The reasons for these characteristics are unknown. Although we found significant LDU differences between bins containing imprinted genes and flanking control bins of equal size, there was no significant enrichment or depletion of any sequence class. We note that we could not explain the higher LDU/Mb ratios at imprinted versus control regions by an increase in gene density in imprinted regions, suggesting that there is no correlation between transcription and meiotic recombination at these regions as seen in other organisms [37–39]. However, hot-spots of recombination within imprinted regions were preferentially associated with genes known to be transcriptionally imprinted compared with other genes in the region. This observation suggests that some epigenetic feature associated with transcriptional imprinting is responsible for this difference.

GC content is the only sequence feature that is correlated positively with LDU/Mb ratios in control regions after correcting for multiple testing. This is in agreement with previous observations that high GC content is a predictor of LD breakdown and implies an association with intense meiotic recombination [16,20,31–34]. There is also only one short DNA motif showing significant positive correlation with LDU/Mb ratios at control regions. Surprisingly, other DNA sequence features are significantly associated with LDU/Mb ratios only at imprinted bins. There are two potential explanations for this unexpected finding: Either there are subtle differences in these sequences at imprinted versus control regions, or these sequences are associated with specific epigenetic modifications that influence the rate of recombination in imprinted regions but not control regions. Epigenetic modifications seem to be the most likely explanation for the heterochiasmy observed at some imprinted regions (Paldi et al. [5], but see also Lercher and Hurst [8] for further discussion) because recombination rate differences between males and females occur even in F_1_ hybrids constructed between inbred strains (http://www.informatics.jax.org) despite the presence of identical DNA sequence between the two sexes.

Little is known about what factors are associated with hot-spots of recombination. For at least a subset of hot-spots, the underlying DNA sequence does not seem to be the main or the only determining factor. For example, a recent comparison between humans and chimpanzees revealed that, despite about 99% identity between the two species at the level of DNA sequence, recombination hot-spots were found rarely at the same positions [15]. Another recent study in human revealed that some recombination hotspots are polymorphic (present/absent) in the absence of local DNA sequence variation between the individuals in which they are present or absent [40]. A possibility to explain this lack of correlation between DNA sequence and patterns of recombination is the involvement of epigenetic factors as major determinants of recombination. Epigenetic factors may vary more substantially across closely related species than DNA sequence. Epigenetic factors are known to play a role in meiotic recombination, but this has so far been demonstrated only in fungi [41,42].

There are several hypotheses that have been proposed to explain heterochiasmy at imprinted loci. Paldi et al. [5] first suggested a model in which differential chromatin remodeling during male and female meiosis associated with epigenetic reprogramming at imprinted chromosomal regions also leads to differential recombination rates between the two sexes. They also stated that imprinted regions have a propensity for higher recombination rates in male meiosis. However, Lercher and Hurst [8] found that most of the imprinted regions (13 out of 16 described at that time) show higher rates of recombination in females than in males. They also found that, in accordance with a theoretical model proposed by Lenormand [43], imprinted regions in which most of the genes are expressed from paternal alleles have higher recombination rates in female meiosis, whereas regions in which most of the genes are maternally expressed show higher recombination rates in male meiosis. Our laboratory has proposed previously that genomic imprinting could be the end result of a complex natural selection process that has operated on differences in the chromatin structure of maternal and paternal chromosomes to facilitate pairing during meiosis and to maintain the distinction between homologs during processes such as DNA repair and recombination in both meiotic and mitotic cells [4,44]. In view of this hypothesis, heterochiasmy could be due to differences in the process of homologous chromosome pairing during male and female meiosis. In mammals, synapsis in male meiosis initiates near the telomeres, and the recombination nodules appear soon after [45,46], whereas in females, interstitially located synaptic initiation sites are also relatively common [47,48]. Possibly some or all synaptic initiation sites are translated into crossovers, resulting in telomeric locations being favored in males more than in females. This hypothesis seems to be in agreement with sex-specific recombination rates published for imprinted chromosomal regions [8]. Most of these regions exhibit higher recombination rates in female meiosis, because they have intermediate position along human chromosomes, whereas most regions with higher recombination rates in male meiosis are located near telomeres or centromeres of human chromosomes. Such meiotic sex differences cannot explain the excess of crossovers at imprinted regions versus flanking regions. However, if imprinted chromosomal regions are active players in the process of synapsis, they have a greater chance to initiate meiotic recombination and so will register a greater number of crossovers in the immediate vicinity.

In this respect, our earlier hypothesis [4] suggests that pairing between homologous chromosomes is facilitated by different epigenetic marks carried by maternal and paternal chromosomes. Given this model, it is curious that the factor exhibiting the strongest correlation with LDU/Mb ratios in imprinted regions is CpG dinucleotides. It is also surprising that the short DNA motif that shows the strongest correlation with LDU/Mb ratios at imprinted (but not control) regions contains a CpG dinucleotide. It is further curious that some prominent steps in the Chromosome 11p15 LD map correspond with or are directly adjacent to imprinted regions that are differentially methylated in somatic cells. We note that these correlations with CpG dinucleotides and regions that are differentially methylated are curious and unexpected because meiotic pairing and recombination take place during prophase of meiosis I, after erasure of parental-specific DNA methylation marks [49]. Given the lack of differential methylation at the time recombination occurs, our model requires that some other epigenetic factor that distinguishes maternal from paternal homologs remain on the chromosomes. There are several studies that suggest that this is, indeed, the case. Despite the absence of differential DNA methylation, parental alleles have been shown to retain an epigenetic memory of their origin during both spermatogenesis and oogenesis [50,51].

Regardless of the underlying mechanism, data accumulated so far supports genomic imprinting as a new source of natural variation in recombination, both between the two sexes and along the chromosomes. Higher rates of recombination at imprinted chromosomal regions might also explain the apparent high incidence of microdeletions recently described at several imprinted loci [52–56].

## Materials and Methods

### Subjects.

DNA samples obtained from unfractionated nucleated peripheral blood cells from the Salt Lake City collection of CEPH/Utah pedigrees (individuals from 45 three-generation families) were studied. All subjects gave informed consent under University of Utah Institutional Review Board–approved protocol number 6090-96.

### Construction of metric LD maps.

LD maps for each region were constructed using LDMAP program as described (http://cedar.genetics.soton.ac.uk/pub/PROGRAMS/LDMAP; [12]) and the data retrieved from the HapMap Project (diplotype data—phase unknown) or from our own linkage analysis data at *IGF2*/*H19* DMR (haplotype data). These maps assign for every SNP two locations, one in kb and one in LDU, based on pairwise association described by the metric ρ = |D|/Q(1 − R), where D is the covariance in a 2 × 2 haplotype table and Q and R are the minor allele frequencies for the given pair of SNPs. For random samples, as in the case of data retrieved from HapMap, ρ equals the maximum value of *D′*.

### Sequence analysis.

CpG islands, defined here as regions of at least 200 nucleotides with minimum 50% C+G content and minimum observed/expected ratios of 0.6, were determined by using the CPGPLOT program in EMBOSS (http://csc-fserve.hh.med.ic.ac.uk/emboss/cpgplot.html). The content in CpG dinucleotides was analyzed by using the CPGREPORT program in EMBOSS (http://genopole.toulouse.inra.fr/bioinfo/emboss/cpgreport.html). Analysis of the other DNA sequence motifs (global GC content and various types of repeats) was performed by using the RepeatMasker program (http://www.repeatmasker.org).

We attempted to determine which sequence classes are responsible for the difference in LDU/Mb between imprinted and control regions (see Tables 1 and 2) by examining sequence classes that have been reported to influence recombination as well as sequence classes that have been tested in similar studies [8,16,32,34,35]. Although Table 1 lists 25 sequence classes that were tested for an effect on LDU/Mb, all classes listed are not independent. We argue that the sequence classes listed in Table 1 comprise no more than eight (and perhaps fewer) independent tests: (1) GC content; (2) CpG dinucleotides and CpG islands; (3) SINE (short interspersed nuclear element) repeats; (4) LINE (long interspersed nuclear element) repeats; (5) LTR (long terminal repeat) elements; (6) DNA elements; (7) small RNAs; and (8) satellites, simple repeats, and low complexity. The classifications “unclassified” repeats, “total interspersed repeats,” and “total repeats” are all interrelated with each other and with classes 3–7. We therefore applied a correction for multiple tests in Table 1 and required a *p-*value of 0.00625 (0.05/8) or lower to reject H_0_: no effect of indicated sequence class on LDU/Mb. For Table 2, we considered six independent tests (the sixth sequence being derived from the first). Therefore, we required a *p-*value of 0.0083 (0.05/6) or lower to reject H_0_: no effect of indicated DNA motif on LDU/Mb ratios.

### Marker genotyping and recombination mapping.

Physical positions of all markers used for this study were obtained from the National Center for Biotechnology Information's MapView. Primers for D11S2071, D11S1363, D11S4046, D11S1318, D11S4088, D11S1883, D11S988, D11S1758, and D11S1760 were all purchased from Invitrogen (former Research Genetics; Carlsbad, California, United States). Genotypes were determined as suggested by the manufacturer. Partial genotyping results were retrieved from the CEPH database (http://www.cephb.fr) for the following markers: WIAF-1667 (rs3216), HRAS1, WIAF-991 (rs3059), MUC5, INS VNTR, TH TaqI, WIAF-3483 (rs1519), WIAF-3839 (rs1875), WIAF-645 (rs2193), WIAF 2696 (rs17081), and WIAF-3649 (rs1685). Recombination events were mapped by pedigree analysis between successive informative markers for which alleles were inherited from different grandparents.

Additional SNPs were genotyped for fine-mapping of recombinants that overlapped the *IGF2/H19* DMR and *KCNQ1OT1* DMR. At *IGF2*/*H19* DMR, mentioned in their physical order from telomere to centromere, we genotyped: rs217243 (CfoI), rs741815 (MspI), rs217233 (RsaI), rs217704 (MspI), rs1635150 (FatI), rs1635153 (RsaI), rs2285935 (MspI), rs217729 (MscI), rs3741216 (MseI), rs2067051 (FspI), rs2075745 (sequencing), rs2075744 (sequencing), rs2839698 (sequencing), rs2525881 (sequencing), rs2251375 (sequencing), rs2251312 (sequencing), rs2158394 (BbvI), rs2071095 (sequencing), rs4930098 (sequencing), rs2107425 (sequencing), rs2071094 (sequencing), rs2735972 (sequencing), rs2735971 (sequencing), rs2735470 (sequencing), rs2735970 (sequencing), rs2525882 (sequencing), rs4930101 (sequencing), rs2525883 (sequencing), rs7105554 (sequencing), rs2735469 (sequencing), rs2525886 (StuI), rs4930103 (sequencing), rs4929983 (sequencing), rs4929984 (sequencing), rs2735467 (sequencing), rs2735461 (BmrI), rs4930110 (sequencing), rs2525887 (sequencing), rs3890907 (sequencing), rs7396803 (sequencing), rs7950715 (sequencing), rs7950932 (AatII), rs7933247 (sequencing), rs3858516 (MspI), rs3858517 (sequencing), rs7125562 (sequencing), rs3858518 (sequencing), rs7950787 (sequencing), rs7107675 (sequencing), rs4384367 (StuI), rs3858520 (sequencing), rs4047059 (sequencing), rs3858521 (sequencing), rs4992750 (sequencing), rs4930125 (MspI), rs7124169 (sequencing), rs7115069 (sequencing), rs4930001 (TaqI), rs7115456 (FokI), rs7103445 (sequencing), rs7130909 (sequencing), rs7119087 (sequencing), rs4930003 (sequencing), rs7924887 (TaqI), rs4930144 (NcoI), rs4930145 (sequencing), rs7106395 (ApaI), rs7928968 (sequencing), rs7940766 (SphI), rs79335743 (sequencing), rs4141121 (sequencing), rs3888172 (NdeII), rs3858522 (sequencing), rs3858523 (FatI), rs3858524 (sequencing), rs3893552 (CfoI), rs7104645 (CfoI), rs7925515 (RsaI), rs7107076 (PstI), rs4930033 (NdeII), and rs7924489 (BfaI). At *KCNQ1OT1* DMR, mentioned in their physical order from telomere to centromere, we genotyped: rs760419 (SacI), rs231357 (HpaII), rs231352 (XbaI), rs231904 (ApaI), rs231847 (HpaII), rs2283202 (HpaII), and rs189161 (ApaI). Genotyping SNPs found at restriction sites was achieved using standard 35-cycle three-step PCR with primers designed to flank the polymorphism and postamplification cleavage with the appropriate restriction endonuclease (all purchased from Roche [Indianapolis, Indiana, United States] or New England Biolabs [Ipswich, Massachusetts, United States]) and using the manufacturer's protocol. Genotyping SNPs found outside any restriction site was achieved using amplification with a high-fidelity DNA polymerase (Platinum Taq Polymerase; Invitrogen), 2% agarose gel electrophoresis, purification using the QIAEX II gel extraction kit (Qiagen, Valencia, California, United States) and subsequent bidirectional sequencing at a sequencing facility.

For linkage analysis at *IGF2*/*H19* DMR, we genotyped in a panel of 45 three-generation families the following ten SNPs in their telomere to centromere order: rs2839704 (RsaI), rs2839702 (AluI), rs2067051 (AatII), rs10732516 (CfoI), rs2525886 (StuI), rs7950932 (AatII), rs3858516 (MspI), rs4384367 (StuI), rs4930001 (TaqI), and rs7924887 (TaqI). Genotyping was achieved as described above.

### Linkage analysis.

Haplotypes at *IGF2*/*H19* DMR were constructed by pedigree analysis. LD between SNP pairs was measured using the absolute value of Lewontin's *D′* where D′ = 1 is reflective of complete LD and 0 corresponds to a state of complete equilibrium [57].

### FGT.

The FGT between each pairwise SNP was performed as previously published [28,29].

### Statistical analysis.

All statistics were generated by using Prism4.0 software (GraphPad).

## Supporting Information

Table S1 Summary of LDU Analysis at Chromosomal Regions Containing Imprinted Genes versus Flanking Control Regions for CEU Population (80 KB DOC)

Table S2 Summary of LDU Analysis at Chromosomal Regions Containing Imprinted Genes versus Flanking Control Regions for YRI Population (80 KB DOC)

Table S3 Summary of LDU Analysis at Chromosomal Regions Containing Imprinted Genes versus Flanking Control Regions for JPT Population (80 KB DOC)

Table S4 Summary of LDU Analysis at Chromosomal Regions Containing Imprinted Genes versus Flanking Control Regions for HCB Population (80 KB DOC)

## Abbreviations

bp: base pairs
CEPH: Centre d'Etude du Polymorphisme Humain
CEU: the CEPH families population with European ancestry
DMR: differentially methylated region
FGT: four-gamete test
HCB: the Han Chinese population in Beijing, China
JPT: the Japanese population in Tokyo, Japan
kb: kilobases
LD: linkage disequilibrium
LDU: linkage disequilibrium units
Mb: megabases
SNP: single nucleotide polymorphisms
YRI: the Yoruba population in Ibadan, Nigeria
