# Supplementary material for: Human Imprinted Chromosomal Regions Are Historical Hot-Spots of Recombination
Source: PLoS Genet. 2006 Jul 7;2(7):e101. doi: 10.1371/journal.pgen.0020101 (PMC1487178; doi:10.1371/journal.pgen.0020101)
Supplement: Supplementary file 2 [file pgen.0020101.st002.doc]

Table S2. Summary of LDU analysis at chromosomal regions containing imprinted genes *versus* flanking control regions for YRI population

| Chromosome | Imprinted gene (genes) | Starting at | Ending to | Total b.p. | Genotyped SNPs | LDU | Informative  SNPs | No blocks | B.p. per block |
| --- | --- | --- | --- | --- | --- | --- | --- | --- | --- |
| **Imprinted regions** | | | | | | | | | |
| 1p36.33 | TP73 | rs950449 | rs4468133 | 999,128 | 1,355 | 56.98 | 273 | 47 | 21,258 |
| 1p31 | NOEY1 | rs12119894 | rs10889781 | 1,002,106 | 1,709 | 38.83 | 356 | 50 | 20,042 |
| 6q24 | PLAGL1 | rs161064 | rs4259269 | 998,452 | 1,488 | 64.802 | 1,115 | 107 | 9,331 |
| 6q25.3 | IGF2R | rs1884187 | rs9457933 | 1,007,455 | 1,801 | 53.496 | 1,171 | 94 | 10,718 |
| 7p12.2 | GRB10 | rs6955602 | rs10230035 | 1,007,508 | 1,482 | 25.23 | 285 | 40 | 25,188 |
| 7q21.3 | CALCR et al. | rs2285507 | rs7794886 | 3,759,486 | 4,778 | 79.10 | 1,144 | 138 | 27,243 |
| 7q32.2 | MEST et al. | rs2727507 | rs10265693 | 1,004,718 | 1,076 | 32.51 | 260 | 47 | 21,377 |
| 11p15.5 | H19 et al. | rs1706879 | rs2239729 | 4,967,439 | 5,745 | 381.69 | 6,418 | 628 | 7,910 |
| 11p13 | WT1 et al. | rs1540318 | rs10835978 | 991,125 | 1,372 | 44.864 | 958 | 73 | 13,577 |
| 13q14.3 | HTR2A | rs259703 | rs9567839 | 995,259 | 1,875 | 37.26 | 348 | 50 | 19,903 |
| 14q32 | DLK1 et al. | rs2753957 | rs11160664 | 1,019,120 | 1,321 | 94.938 | 992 | 139 | 7,332 |
| 15q11-q13 | MKRN3 et al. | rs4778545 | rs7178713 | 3,240,538 | 4,889 | 205.30 | 3,616 | 322 | 10,064 |
| 18q21.1 | ELONGIN A2 | rs1377016 | rs12604167 | 996,300 | 1,455 | 26.25 | 473 | 45 | 22,140 |
| 19q13.43 | PEG3 | rs8100287 | rs8110400 | 985,981 | 1,275 | 43.45 | 276 | 43 | 22,930 |
| 20q11.2 | NNAT | rs6017076 | rs6022419 | 989,019 | 1,898 | 21.74 | 259 | 32 | 30,907 |
| 20q13.12 | L3MBTL | rs2223557 | rs6031301 | 997,166 | 1,825 | 30.16 | 269 | 51 | 19,552 |
| 20q13.32 | GNAS | rs6513380 | rs197820 | 994,148 | 1,740 | 36.28 | 274 | 48 | 20,711 |
| Total | | | | 25,954,948 | 37,084 | 1,272.88 | 18,487 | 1,954 | 13,283* |
| **Control regions** | | | | | | | | | |
| 1p36.33 | - | rs2985859  rs4468133 | rs950449  rs6658098 | 996,327 | 1,777 | 46.78 | 296 | 55 | 18,115 |
| 1p31 | - | rs1885277  rs10889781 | rs12119894  rs1567214 | 1,000,792 | 1,059 | 23.98 | 344 | 35 | 28,594 |
| 6q24 | - | rs12201873  rs4259269 | rs161064  rs7742978 | 1,006,235 | 1,731 | 46.435 | 1,222 | 86 | 11,700 |
| 6q25.3 | - | rs3117148  rs9457933 | rs1884187  rs3798906 | 997,665 | 1,859 | 52.663 | 1,386 | 103 | 9,686 |
| 7p12.2 | - | rs1365086  rs10230035 | rs6955602  rs7803298 | 1,012,520 | 1,280 | 19.49 | 249 | 27 | 37,501 |
| 7q21.3 | - | rs10228442  rs7794886 | rs2285507  rs3801241 | 3,759,338 | 3,389 | 67.99 | 956 | 125 | 30,075 |
| 7q32.2 | - | rs6971551  rs10265693 | rs2727507  rs1619267 | 1,007,687 | 1,131 | 18.25 | 252 | 34 | 29,638 |
| 11p15.5 | - | rs3741411  rs2239729 | rs1706879  rs7123728 | 4,970,820 | 8,034 | 276.42 | 4,318 | 458 | 10,853 |
| 11p13 | - | rs1323738  rs10835978 | rs1540318  rs266461 | 997,876 | 867 | 20.637 | 549 | 36 | 27,719 |
| 13q14.3 | - | rs9534241  rs9567839 | rs259703  rs6561405 | 1,005,407 | 1,676 | 22.59 | 366 | 43 | 23,382 |
| 14q32 | - | rs941900  rs11160664 | rs2753957  rs2145680 | 1,020,828 | 799 | 33.05 | 540 | 56 | 18,229 |
| 15q11-q13 | - | rs7162091  rs7178713 | rs4778545  rs3932767 | 3,138,107 | 2,942 | 164.14 | 2,106 | 229 | 13,704 |
| 18q21.1 | - | rs920295  rs12604167 | rs1377016  rs8093883 | 1,003,344 | 1,169 | 35.86 | 455 | 59 | 17,006 |
| 19q13.43 | - | rs412676  rs8110400 | rs8100287  rs13345819 | 993,138 | 1,327 | 40.45 | 302 | 48 | 20,690 |
| 20q11.2 | - | rs220076  rs6022419 | rs6107076  rs805557 | 1,005,442 | 1,423 | 22.95 | 241 | 33 | 30,468 |
| 20q13.12 | - | rs6072770  rs6031301 | rs2223557  rs3092749 | 996,221 | 2,362 | 39.3 | 272 | 51 | 19,534 |
| 20q13.32 | - | rs6025863  rs197820 | rs6513380  rs6070977 | 992,883 | 2,254 | 50.18 | 291 | 52 | 19,094 |
| Total | | | | 25,904,630 | 35,079 | 981.125 | 14,145 | 1,530 | 16,931* |
| Paired *t* test (*P* value) | | | | 0.4518 | 0.5950 | 0.0284 | 0.1061 | 0.0478 | 0.0306 |

* mean haplotype size
