# Supplementary material for: Human Imprinted Chromosomal Regions Are Historical Hot-Spots of Recombination
Source: PLoS Genet. 2006 Jul 7;2(7):e101. doi: 10.1371/journal.pgen.0020101 (PMC1487178; doi:10.1371/journal.pgen.0020101)
Supplement: Supplementary file 3 [file pgen.0020101.st003.doc]

Table S3. Summary of LDU analysis at chromosomal regions containing imprinted genes *versus* flanking control regions for JPT population

| Chromosome | Imprinted gene (genes) | Starting at | Ending to | Total b.p. | Genotyped SNPs | LDU | Informative SNPs | No blocks | B.p. per block |
| --- | --- | --- | --- | --- | --- | --- | --- | --- | --- |
| **Imprinted regions** | | | | | | | | | |
| 1p36.33 | TP73 | rs950449 | rs4468133 | 999,128 | 1,355 | 44.64 | 263 | 52 | 19,124 |
| 1p31 | NOEY1 | rs12119894 | rs10889781 | 1,002,106 | 1,709 | 25.72 | 310 | 39 | 25,695 |
| 6q24 | PLAGL1 | rs161064 | rs4259269 | 998,452 | 1,488 | 40.619 | 747 | 73 | 13,677 |
| 6q25.3 | IGF2R | rs1884187 | rs9457933 | 1,007,455 | 1,801 | 20.571 | 297 | 28 | 35,981 |
| 7p12.2 | GRB10 | rs6955602 | rs10230035 | 1,007,508 | 1,482 | 20.08 | 262 | 35 | 28,786 |
| 7q21.3 | CALCR et al. | rs2285507 | rs7794886 | 3,759,486 | 4,778 | 57.85 | 1,011 | 108 | 34,810 |
| 7q32.2 | MEST et al. | rs2727507 | rs10265693 | 1,004,718 | 1,076 | 15.18 | 208 | 25 | 40,189 |
| 11p15.5 | H19 et al. | rs1706879 | rs2239729 | 4,967,439 | 5,745 | 112.794 | 1,362 | 176 | 28,224 |
| 11p13 | WT1 et al. | rs1540318 | rs10835978 | 991,125 | 1,372 | 13.976 | 225 | 29 | 34,177 |
| 13q14.3 | HTR2A | rs259703 | rs9567839 | 995,259 | 1,875 | 23.42 | 315 | 38 | 26,191 |
| 14q32 | DLK1 et al. | rs2753957 | rs11160664 | 1,019,120 | 1,321 | 40.624 | 352 | 56 | 18,199 |
| 15q11-q13 | MKRN3 et al. | rs4778545 | rs7178713 | 3,240,538 | 4,889 | 74.483 | 722 | 102 | 31,770 |
| 18q21.1 | ELONGIN A2 | rs1377016 | rs12604167 | 996,300 | 1,455 | 26.75 | 463 | 44 | 22,643 |
| 19q13.43 | PEG3 | rs8100287 | rs8110400 | 985,981 | 1,275 | 21.04 | 241 | 31 | 31,806 |
| 20q11.2 | NNAT | rs6017076 | rs6022419 | 989,019 | 1,898 | 12.34 | 237 | 15 | 65,935 |
| 20q13.12 | L3MBTL | rs2223557 | rs6031301 | 997,166 | 1,825 | 22.09 | 239 | 35 | 28,490 |
| 20q13.32 | GNAS | rs6513380 | rs197820 | 994,148 | 1,740 | 23.42 | 234 | 40 | 24,854 |
| Total | | | | 25,954,948 | 37,084 | 595.597 | 7,488 | 926 | 28,029* |
| **Control regions** | | | | | | | | | |
| 1p36.33 | - | rs2985859  rs4468133 | rs950449  rs6658098 | 996,327 | 1,777 | 29.48 | 282 | 45 | 22,141 |
| 1p31 | - | rs1885277  rs10889781 | rs12119894  rs1567214 | 1,000,792 | 1,059 | 20.32 | 250 | 29 | 34,510 |
| 6q24 | - | rs12201873  rs4259269 | rs161064  rs7742978 | 1,006,235 | 1,731 | 15.15 | 458 | 29 | 34,698 |
| 6q25.3 | - | rs3117148  rs9457933 | rs1884187  rs3798906 | 997,665 | 1,859 | 10.045 | 343 | 23 | 43,377 |
| 7p12.2 | - | rs1365086  rs10230035 | rs6955602  rs7803298 | 1,012,520 | 1,280 | 13.84 | 223 | 25 | 40,501 |
| 7q21.3 | - | rs10228442  rs7794886 | rs2285507  rs3801241 | 3,759,338 | 3,389 | 46.65 | 853 | 78 | 48,197 |
| 7q32.2 | - | rs6971551  rs10265693 | rs2727507  rs1619267 | 1,007,687 | 1,131 | 19.60 | 228 | 28 | 35,989 |
| 11p15.5 | - | rs3741411  rs2239729 | rs1706879  rs7123728 | 4,970,820 | 8,034 | 99.536 | 1,176 | 162 | 30,684 |
| 11p13 | - | rs1323738  rs10835978 | rs1540318  rs266461 | 997,876 | 867 | 2.276 | 209 | 9 | 110,875 |
| 13q14.3 | - | rs9534241  rs9567839 | rs259703  rs6561405 | 1,005,407 | 1,676 | 21.05 | 340 | 38 | 26,458 |
| 14q32 | - | rs941900  rs11160664 | rs2753957  rs2145680 | 1,020,828 | 799 | 12.316 | 219 | 30 | 34,028 |
| 15q11-q13 | - | rs7162091  rs7178713 | rs4778545  rs3932767 | 3,138,107 | 2,942 | 47.635 | 488 | 67 | 46,837 |
| 18q21.1 | - | rs920295  rs12604167 | rs1377016  rs8093883 | 1,003,344 | 1,169 | 17.89 | 394 | 35 | 28,667 |
| 19q13.43 | - | rs412676  rs8110400 | rs8100287  rs13345819 | 993,138 | 1,327 | 28.74 | 286 | 41 | 24,223 |
| 20q11.2 | - | rs220076  rs6022419 | rs6107076  rs805557 | 1,005,442 | 1,423 | 14.17 | 163 | 26 | 38,671 |
| 20q13.12 | - | rs6072770  rs6031301 | rs2223557  rs3092749 | 996,221 | 2,362 | 22.48 | 225 | 33 | 30,189 |
| 20q13.32 | - | rs6025863  rs197820 | rs6513380  rs6070977 | 992,883 | 2,254 | 28.84 | 258 | 47 | 21,125 |
| Total | | | | 25,904,630 | 35,079 | 450.018 | 6,395 | 745 | 34,771* |
| Paired *t* test (*P* value) | | | | 0.4518 | 0.5950 | 0.0058 | 0.0196 | 0.0139 | 0.1222 |

* mean haplotype size
